# Supplementary material for: TVA-based modeling of short-term memory capacity, speed of processing and perceptual threshold in chronic stroke patients undergoing cognitive training: case-control differences, reliability, and associations with cognitive performance
Source: PeerJ. 2020 Oct 28;8:e9948. doi: 10.7717/peerj.9948 (PMC7602688; doi:10.7717/peerj.9948)
Supplement: Supplemental Information 1 — *Significant after Bonferroni correction. K = short-term memory capacity. C = perceptual processing speed. t0 = perceptual threshold. Estimate = unstandardized regression coefficients b. std.error = standard error. t = t-value. p = p-value. Cohen’s D was calculated using two times the t-value divided by the square root of the degrees of freedom. [file peerj-08-9948-s001.docx]

| Parameter | | group | age | sex | education |
| --- | --- | --- | --- | --- | --- |
| *K* | estimate (std.error) | -0.283 (0.109) | -0.02 (0.005) | -0.138 (0.105) | 0.007 (0.015) |
|  | *t (p)* | -2.586 (0.01)* | -3.707 (<.001)* | -1.317 (0.19) | 0.49 (0.625) |
|  | Cohen's d | -0.37 | -0.531 | -0.189 | 0.07 |
| *C* | estimate (std.error) | -5.969 (2.114) | -0.316 (0.103) | 2.671 (2.022) | 0.405 (0.285) |
|  | *t (p)* | -2.823 (0.005)* | -3.083 (0.002)* | 1.321 (0.188) | 1.419 (0.157) |
|  | Cohen's d | -0.404 | -0.442 | 0.189 | 0.203 |
| *t*_0_ | estimate (std.error) | 6.827 (2.605) | 0.395 (0.126) | -7.125 (2.491) | -0.129 (0.352) |
|  | *t (p)* | 2.621 (0.009)* | 3.13 (0.002)* | -2.86 (0.005)* | -0.366 (0.715) |
|  | Cohen's d | 0.375 | 0.448 | -0.41 | -0.052 |
| ErrorRate | estimate (std.error) | -0.023 (0.013) | 0 (0.001) | -0.006 (0.013) | -0.002 (0.002) |
|  | *t (p)* | -1.737 (0.084) | -0.011 (0.991) | -0.449 (0.654) | -1.096 (0.275) |
|  | Cohen's d | -0.249 | -0.002 | -0.064 | -0.157 |

**Suppl. Table 1.** **Summary statistics for the linear models testing for association between TVA parameters (dependent variables) and group (case-control comparisons), including age, sex and education as covariates.** *Significant after Bonferroni correction. K = short-term memory capacity. C = perceptual processing speed. t_0_ = perceptual threshold. Estimate = unstandardized regression coefficients b. std.error = standard error. t = t-value. p = p-value. Cohen’s D was calculated using two times the t-value divided by the square root of the degrees of freedom.
